# BRCA and Early Events in the Development of Serous Ovarian Cancer

**DOI:** 10.3389/fonc.2014.00005

**Published:** 2014-01-23

**Authors:** Sophia H. L. George, Patricia Shaw

**Affiliations:** ^1^Department of Laboratory Medicine and Pathobiology, Campbell Family Institute for Breast Cancer Research at Princess Margaret Cancer Centre, University Health Network, University of Toronto, Toronto, ON, Canada

**Keywords:** BRCA, fallopian tube epithelium, high-grade serous carcinoma

## Abstract

Women who have an inherited mutation in the BRCA1 or BRCA2 genes have a substantial increased lifetime risk of developing epithelial ovarian cancer (EOC), and epidemiological factors related to parity, ovulation, and hormone regulation have a dramatic effect on the risk in both BRCA mutation carriers and non-carriers. The most common and most aggressive histotype of EOC, high-grade serous carcinoma (HGSC), is also the histotype associated with germline BRCA mutations. In recent years, evidence has emerged indicating that the likely tissue of origin of HGSC is the fallopian tube. We have reviewed, what is known about the fallopian tube in BRCA mutation carriers at both the transcriptional and translational aspect of their biology. We propose that changes of the transcriptome in BRCA heterozygotes reflect an altered response to the ovulatory stresses from the microenvironment, which may include the post-ovulation inflammatory response and altered reproductive hormone physiology.

## Introduction

In 2013, about 22,240 women in the United States would have been diagnosed with invasive epithelial ovarian cancer (EOC) and an estimated 14,000 women with EOC would have died (1). There are five major histotypes of EOC and they are distinct epidemiologically, phenotypically, and molecularly, namely: mucinous, endometrioid, clear cell, low-grade serous, and high-grade serous carcinoma (HGSC). Of these, HGSC is the most prevalent histotype in the Western Hemisphere, the most lethal, typically is diagnosed at an advanced stage, and there are no effective cancer screening strategies. More than 75% of women with this diagnosis will succumb to the disease after combined first line treatment, which includes surgery and adjuvant platinum-based chemotherapy, with a 5-year survival of <30% (1, 2). HGSC is a genetically unstable tumor, characterized by a varied histomorphology unified by marked pleomorphism, a high mitotic rate, and biomarker expression reflective of the most common molecular alterations. The latter includes the near ubiquitous presence of a mutation in the tumor suppressor p53 (TP53), resulting in either over accumulation of p53 protein by immunohistochemistry (missense – 60% of analyzed cases) or complete loss of protein expression (frameshift/splicing junctions/non-sense – 39% of analyzed cases) (3). Mutations of p53 are present in early stage HGSC, and mutant TP53 is likely an essential driver mutation required for the early pathogenesis of HGSC (4). Other recurrent mutations in HGSC are infrequent, but most prominently include BRCA1 and BRCA2, with BRCA germline mutations seen in 13–16% (5), and somatic mutations seen in about 6% of cases.

High-grade serous carcinoma is the predominant histotype associated with hereditary breast-ovarian cancer (6, 7). Women with inherited mutations of BRCA1 or BRCA2, have a lifetime risk of 40–60% (BRCA1) and 11–27% (BRCA2) (8–12). Women known to be at increased genetic risk based on family history and/or genetic testing are offered risk-reducing salpingo-oophorectomy (RRSO), which reduces the risk of malignancy by up to 96% (13, 14) and is usually performed after completion of childbearing and while the woman is still pre-menopausal (13, 15). An unexpected finding on histopathology review of the resected fallopian tubes in this population was the presence of clinically undetected, occult carcinomas in the fallopian tubes, a tissue previously thought to develop carcinomas only rarely. These were seen more frequently than in the ovarian tissues (16). This discovery was followed by careful review of the fallopian tube tissues, and subsequent studies have reported histological lesions purported to be HGSC precursors in the fallopian tube epithelium – these had not been found in the genetic high-risk ovarian tissues (16–22). Hence, detailed histo-pathological examination of the resected ovaries and fallopian tubes in BRCA mutation carriers has led to a radical change in existing paradigms of serous carcinogenesis. Because loss of BRCA function is frequent in HGSC, study of the effect of BRCA, including heterozygosity/haploinsufficiency and loss of function in the fallopian tube epithelium prior to the development of HGSC, offers opportunities to better understand HGSC pathogenesis, and should lead to the development of novel and more effective preventative, and possibly, screening strategies.

## BRCA1 and BRCA2 and High-Grade Serous Cancer

Molecularly, the breast cancer susceptibility genes (BRCA) BRCA1 and BRCA2 can sense DNA damage and are involved in DNA repair via interactions with RAD51 (23–25); these three proteins are essential for genomic stability in normal cells predominantly through the homologous recombination pathway (HR) (26). BRCA1 is a known modulator of the cell-cycle at the G2-M checkpoint (27) operating through co-activation with p53 (28) and has also been shown to epigenetically regulate the oncogenic microRNA 155 and to maintain heterochromatin structure via ubiquitylation of H2A (29, 30). Inherited mutations in BRCA1/BRCA2 confer an autosomal-dominant effect and range from being deleterious to protein function to being of uncertain significance (31).

Breast cancer susceptibility gene mutation carriers develop cancers in hormonally regulated tissues, most frequently in breast and ovarian/tubal tissues, but a unifying mechanism of early malignant transformation in these tissues is not known. The BRCA associated carcinomas share some common features including a high-grade phenotype, frequent mutations of TP53, and other copy number landscape features like Cyclin-E amplification and deletion of Rb (32). Altered BRCA function in HGSC does not only occur in the setting of hereditary disease. Dysfunction of BRCA1 or BRCA2 is prevalent in patients with HGSC via 6% somatic mutations (5, 33–35); 13–31% promoter hypermethylation (5, 36–38); 7.9–17% amplification of EMSY (5, 39, 40); or 13.2% promoter hypermethylation of FANCF (41). The sum of these genomic alterations predominantly in the HR pathway of HGSC has led to determining the “BRCAness” profile in patients (42, 43). BRCAness is defined as a phenotype determined by deficiencies in the double strand break (DSB) repair pathways, as seen in tumors associated with germline BRCA mutations and a subset of sporadic high-grade serous ovarian cancers. An understanding of the early molecular changes in genetic high-risk patients may therefore also be of importance to many of the sporadic cancers. Patients with the BRCAness profile most likely will benefit from treatments affecting other DNA repair pathways – specifically PARP inhibitors (43). Outcome data suggests that patients with loss of function of BRCA have improved survival, but recently a study by McLaughlin and colleagues determined that although BRCA mutation carriers have a short-term (up to 5 years post diagnosis) benefit and response to platinum-based therapy, there is a lack of long-term (up to 10 years post diagnosis) survival benefit (44). Most promisingly, the loss of function of BRCA1/BRCA2 whether genetic or epigenetic by mechanisms including promoter hypermethylation, offers the possibility of improved therapies with poly (ADP-ribose) polymerase (PARP) inhibitors (43).

## BRCA1 and BRCA2 and the Fallopian Tube Epithelium

**Figure 1:**
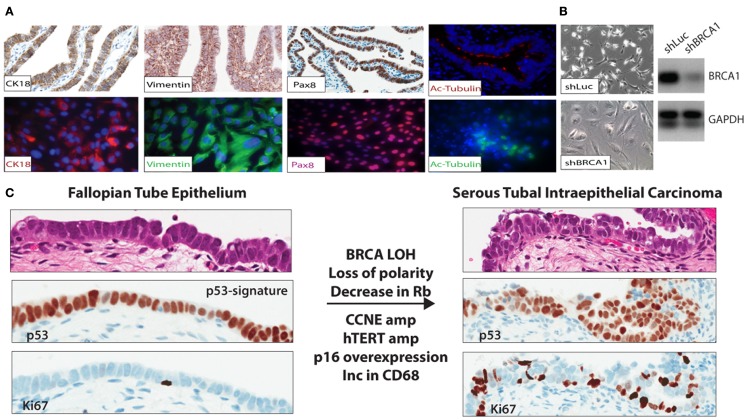
**(A)** FTE cell lines were established to study gene specific effects in relation to BRCA abrogation in BRCA mutation carriers and other aberrations identified in the precursor lesions and malignant lesions observed *in situ* in the distal end of the FTE. **(B)** FTE cell lines established from normal FTE tissue were infected with a short hairpin to BRCA1 (shBRCA1). The FTE cells with BRCA loss have the classic phenotype of senescent cells – flat, enlarged, and vacuolated. PCR confirmed knockdown. **(C)** In the p53 signature in the normal FTE, low proliferation, normal cell polarity, and over-expression of p53 are observed. Thus far, BRCA loss-of-heterozygosity (in mutation carriers), decrease in Rb, and increase in p16 (immunohistochemistry), CCNE1 amplification, and over-expression (FISH and immunohistochemistry); hTERT amplification (FISH), common in HGSC are also observed in the STIC lesions.

The mechanisms underlying malignant transformation in these estrogen responsive tissues are poorly understood, but likely involve loss of heterozygosity of the remaining wild type BRCA allele (45) in addition to inactivation of p53. During ovulation, it is thought that high levels of reactive oxygen species (ROS) are released via the cytokine surge accompanied with lysis of the ovum (follicular fluid). These species have a complex role in the development and progression of cancer (46). The high ROS levels are likely a source of “carcinogens,” which cause DNA damage in the FTE and possibly contribute to the mutations in TP53. In normal cells repair of DNA damage results in cell-cycle arrest through senescence or death as demonstrated in epithelial cell lines established from FTE (Figures 1A,B). This process must be overcome for transformation to occur (47). In high-grade serous ovarian cancer cells, 99% of tumors have a mutation in TP53, indicating that the mutation likely occurs early in disease progression (3, 5). This combination – TP53 mutation and BRCA loss, can provide an escape or by-pass through the cell-cycle checkpoints to allow additional cancer promoting mutations, amplifications, or deletions. Therefore, BRCA1/BRCA2 deficient cells [lacking ATM/ATR-CHK2 pathway (48)] cannot sense DNA damage in order to transduce signal to the already TP53 mutant cells. In this setting, cells can overcome the barriers for cell-cycle progression, however this may not be sufficient for transformation into a tumor.

In normal cells of mutation carriers, only one allele is mutated, and BRCA1 function is presumed to be intact. This may however not be true, as evidence in support of BRCA1 haploinsufficiency accumulates. For example, in normal human mammary epithelial cells from BRCA1 heterozygotes, DNA homologous repair is suppressed (49). BRCA1 haploinsufficiency may be an early but not a sufficient step of BRCA1-mediated breast carcinogenesis. In HGSC, it is uncertain when during malignant transformation of FTE, loss of BRCA1 function occurs. In contrast to breast cancer, it seems likely altered p53 function resulting from p53 mutation occurs prior to loss of the wild type BRCA1 allele in FTE transformation. Loss of BRCA1 protein and loss of heterozygosity is seen once malignant transformation has occurred but, according to Norquist et al. not in early precancerous lesions (45). The p53 mutation is thought to promote genomic instability, a hallmark of high-grade serous cancer, and cooperates with BRCA1 loss or a dysfunctional HR pathway to mediate the extent of genomic amplifications and gains so commonly seen in HGSC.

### p53 signature and serous tubal intraepithelial carcinoma

For many years, in the absence of a reproducible histological precursor lesion of HGSC, the cell of origin was presumed to be the ovarian surface epithelium (OSE), a modified type of mesothelium. Detailed histo-pathological examination of tubal epithelia (FTE) in the genetically high-risk population undergoing risk-reducing surgery has led to the discovery of putative cancer precursor lesions in the fallopian tube, some of which, i.e., the p53 signature – described as a string of 10–12 histologically normal secretory (non-ciliated) cells expressing the TP53 protein with a low proliferation rate (Ki67) (50), are found with a similar frequency in BRCA mutation carriers and non-carriers. Two independent studies reported similar findings albeit at different frequencies of p53-signatures between the two study cohorts: 11 and 19% (51) and 24 and 33% (52) in women with germline BRCA mutations and population control, respectively. The cells within the p53 signature are Pax8 positive and up-regulate phosphorylated – γH2AX, reflective of concomitant DNA damage. Women with an inherited mutation in the TP53 gene – the Li Fraumeni syndrome, have an increased risked in developing between five and six different cancers (breast, brain, soft tissue sarcomas, and blood cancers) throughout their lifetime (52). These patients, however, do not have an increased incidence of developing high-grade serous ovarian cancer, but have an increased number of p53-signatures compared to the rest of the population. In addition, in a small epidemiological study, p53-signatures were not associated with the traditional risk factors of breast-feeding, parity and tubal ligation, bringing into questions whether the p53 signature is a true cancer precursor lesion (53). However, it can be said that loss of normal p53 function is necessary, but not sufficient to promote carcinogenesis of epithelial cells in the distal fallopian tube.

Occult invasive carcinoma and serous tubal intraepithelial carcinomas (STICs) were identified in the fallopian tubes of mutation carriers undergoing risk-reducing surgery, with an incidence of about 4–6% for occult cancers (16, 54, 55). Importantly, STICs are found not only in BRCA mutation carriers, but are also detected in about 60% of sporadic HGSC (19, 56). STICs are thought to have progressed from the p53 signature and are characterized as being highly proliferative (>10% Ki67) (57), show loss of apical to basal nuclear polarity and, in common with HGSC, demonstrate: over-expression of cyclin-E (58), amplification of hTERT (59), p16 over-expression (CDKN2A), loss of Retinoblastoma protein (Rb) (60), and up-regulation of the PI3K pathway (61) (Figure 1C). In mutation carriers undergoing RRSO, STICs were identified in at least 8% of cases, a higher frequency than seen in patients at low genetic risk (51, 52, 62, 63).

Like HGSC, the frequency of STIC lesions increases with age, is increased in BRCA1/2 mutation carriers, and is lower with oral contraceptive use, all features providing further evidence that STIC is an immediate precursor of invasive and clinically detectable carcinoma (53). These intraepithelial carcinomas should not be considered as only *in situ* carcinomas, because in at least some cases while tumor cells do not invade underlying stroma, they can detach, and because of the accessibility of the ipsilateral ovary and other peritoneal surfaces to the tubal fimbria, cells may implant and establish tumor growth in other sites. Currently, little evidence exists that patients with only a diagnosis of STIC require adjuvant therapy (64). Further molecular and genetic characterization of STIC is ongoing, but molecular evidence to date indicates that alterations commonly seen in HGSC are also present in STIC. Lesions that precede the STIC, are not well characterized, but currently the term serous tubal intraepithelial lesion (STIL) is given to lesions according to criteria recommended in a proposed diagnostic algorithm. The STIL is described as a lesion, which has abnormal p53 expression by immunohistochemistry and increased proliferation relative to background (tubal epithelium) but <10% Ki67 positive. (57, 65). Ongoing studies are required to further define this lesion as current definitions lack diagnostic reproducibility. Other than the changes associated with the p53 signature, molecular changes which precede the establishment of an intraepithelial cancer are not well documented. Indeed, these lesions are uncommon, and identified with poor reproducibility.

### Normal tube epithelium in BRCA mutation carriers

Hormonally responsive epithelia, from breast, ovary, and fallopian tube, are the preferential targets for malignant transformation in BRCA1/2 mutation carriers and the mechanisms are increasingly being determined primarily through studying breast epithelia (66). Evidence is emerging nonetheless that morphologically normal fallopian tube epithelium from women with inherited mutations, differs significantly from the tubal epithelium of women at low cancer risk. Differences in morphologically normal epithelium from BRCA mutation carriers have shed light into the effects of heterozygosity and predisposition to high-grade serous ovarian cancer. In five previous reports, we have used morphologically normal fallopian tubal epithelium from BRCA1 and BRCA 2 (FTE-BRCA) mutation carriers and non-mutation carriers (FTE-normal), to compare gene expression profiles to identify differences conferred by the presence of one mutant allele (67–71). In addition to family history, a major risk factor is number of lifetime ovulations, and oral contraception use and increase in parity lead to a reduction in EOC risk (72). The formerly prevailing incessant ovulation hypothesis first described by Fathalla suggested that continuous disruption and surrounding inflammation of the OSE during ovulation led to the development of carcinoma in the ovary (73). It is likely however that the effects of ovulation are still important in malignant transformation, but the effects are on fimbrial, not ovarian, epithelium.

Therefore in the design of experiments, the patient tissues analyzed were controlled for not only age and menopause but also stage in the ovarian cycle – follicular (proliferative phase) and luteal (post-ovulatory phase) at the time of surgery (70, 71). We showed that the BRCA mutation in morphologically normal fallopian tube epithelium confers a significantly altered gene expression signature. Some of these altered pathways include the TGF-β pathway, MAP kinase pathway, the adipokine signaling pathway, inflammatory pathway, and the p53-signaling pathway (70, 71). In particular genes involved in DNA damage and inflammation were validated as both having transcriptional and translational differential expression in the normal fallopian tubes (ampulla and fimbria) of BRCA mutation carriers. Namely, DAB, NAMPT, C/EBP-δ, GADD45β, and NF-κB are genes involved in the Jak/Stat, DNA damage, and TGF-β pathways and are prominently differentially expressed in mutation carriers and in HGSC. In these studies, we noted, that BRCA mRNA levels were not substantially different between carriers and non-carriers, indicating that the wild type allele was still intact. In an independent study, Press et al. reported significant differences in proliferation and cell-cycle regulation in BRCA mutation carriers (with and without occult carcinoma) (74).

**Figure 2:**
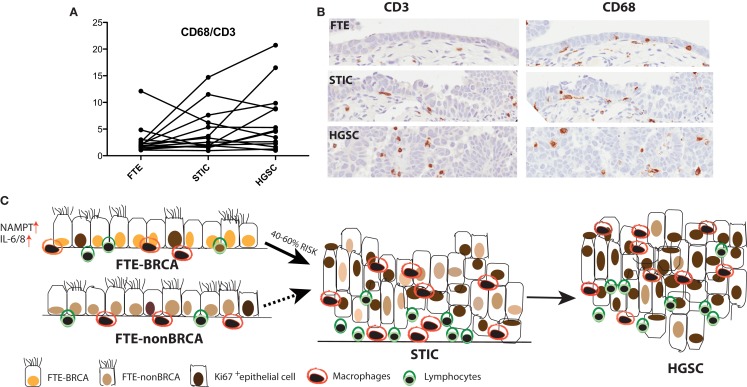
**(A)** A trend is observed, where in some cases the ratio of CD68^+^ macrophages to CD3^+^ lymphocytes increases from the normal FTE to STIC to the concomitant cancer. **(B)** Immunohistochemistry of CD3^+^ and CD68^+^ in normal FTE, STIC (same STIC as depicted in Figure 1), and HGSC. **(C)** High-grade serous carcinoma is the most common type of ovarian cancer and women with BRCA1/2 mutations have a 40–60% increased lifetime risk for developing the disease. Interrogation of the normal FTE microenvironment demonstrates that there is no inherent difference in proliferation or in some immune cell populations within the histological normal tissue in FTE of BRCA1/2 mutation carriers compared to the normal population. An increase in proliferation and lymphocytes and macrophages occurs later in tumor progression when the FTE have already lost cell-cycle progression barriers and there is histological evidence of a precursor lesion.

We subsequently analyzed the distal end FTE (the fimbria), the anatomical region of highest risk and the ampulla for: (1) the presence of immune infiltrates (CD3^+^ and CD8^+^ lymphocytes and CD68^+^ macrophages) and (2) the proliferation status of FTE cells in both BRCA mutation carriers and population control. This study although not exhaustive, revealed that independent of BRCA mutation status: (1) macrophages were more prevalent in the luteal phase than the follicular phase of the ovarian cycle and (2) proliferation in FTE cells is predominantly an effect of the follicular phase rather than BRCA mutation status in histologically normal tissue (Figure 2). However, a small subset of FTEs from BRCA2 mutation carriers had a diffuse increase in proliferation in the absence of histological lesions, but overall there was no statistical difference in proliferation compared to the control tissues (68). Therefore, we propose that chronic inflammatory states through cyclical ovulation and the presence of a mutated BRCA allele can predispose the normal FTE to develop lesions, which may lead to serous carcinoma. We hypothesize that this occurs through deregulation of DNA damage response genes and synergistically through up-regulation of cytokines, pro-inflammatory, and proliferation genes. It is possible that changes demonstrated in gene expression profiles reflect the earliest alterations in cancer development, and/or that they are markers of increased cancer risk.

## Ovulatory Cycle and BRCA in the Fallopian Tube Epithelium

Most women who develop sporadic cases of EOC are peri- or post-menopausal with a mean age of 58 years (75); however, BRCA1 mutation carriers develop the disease earlier with a mean age of 51 years and BRCA2 mutation carriers a bit later, with a mean age of 57 years (75–78). In addition to family history, the major epidemiological risk factors for EOC indicate a strong influence of reproductive factors and reproductive hormones. Risk factors including nulliparity, early age of menarche, late age of menopause, hormone replacement, obesity, and protective factors including oral contraceptive use, indicate an association with increased lifetime ovulations and/or greater lifetime exposure to estrogen. A higher risk of ovarian cancer has been reported with cyclical use of hormone replacement therapy rather than continuous use or any use of estrogen or progestin after menopause (79) for both BRCA mutation carriers and non-carriers (72).

The influence of sex hormones on tubal/ovarian malignant transformation is not well understood, but seems likely that the BRCA1/2 associated changes in reproductive hormones and their receptors play a role in tumor formation, in addition to the alterations in DNA damage repair. BRCA1/2 mutation carriers do not have menopause at an early age (80). Higher circulating estradiol is associated in the general population with a pre-menopausal breast cancer risk, and BRCA2 carriers with breast cancer do have higher estradiol levels in the early follicular phase, but a similar association with circulating progesterone is not seen. It has recently been shown that mutation carriers have higher levels of both estradiol and progesterone during the luteal, not follicular phase, leading the investigators to suggest a defect in steroid hormone regulation potentiates the mutagenic effect of the BRCA mutation (80, 81). In mice, it has been shown that granulosa cells in mice lacking functional Brca1 are exposed to increased estradiol stimulation due to a combination of a prolonged pre-ovulatory (proestrus) phase of the estrus cycle and increased levels of circulating estradiol. In addition, estrogen biosynthesis in granulosa cells is altered in mice not only with a deleterious homozygous mutation but also in mice with a heterozygous Brca1 mutation (82), a state which mimics the BRCA1 mutation carriers. This provides further evidence that heterozygous BRCA1 mutations are associated with phenotypic changes.

**Figure 3:**
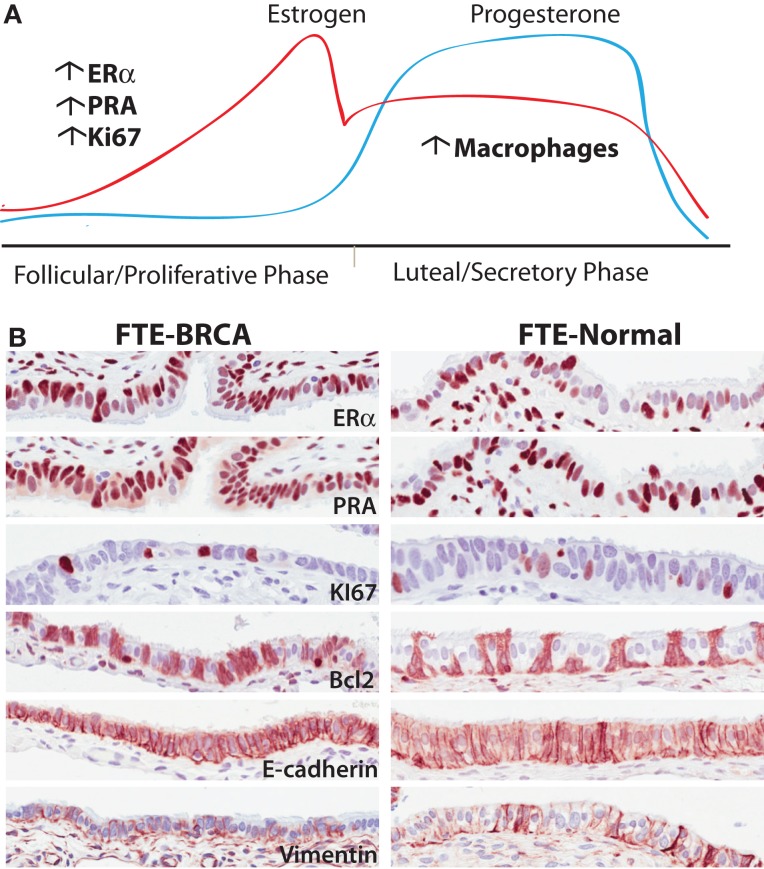
**(A)** The different phases of the ovarian cycle in pre-menopausal women is the dominant effect on gene transcription in epithelia of normal fallopian tubes in BRCA mutation carriers. Translationally, there are more cells expressing ER, PR in the follicular phase of the cycle independent of mutation status. Similarly there are significantly more cells responding to the mitogenic effects of estrogen observed by an increase in proliferation. In the post-ovulatory phase, there is an observed increase in macrophages in both BRCA mutation carriers and non-carriers. **(B)** Representative images of proteins expressed in normal FTE-BRCA and FTE-normal that seemingly look and express these proteins similarly. Underlying these morphological similarities is a potential haploinsufficiency predisposing FTE-BRCA to cytotoxic stresses.

The role of estrogen and progesterone in early malignant transformation in the FTE is not yet well understood. Estrogen mediates its action primarily through the estrogen receptor (ERα and ERβ). Estrogen stimulates the expression of a number of genes that promote cell proliferation, motility/invasion, and inhibition of apoptosis namely: IL6, TGF-α, EGF, PI3K/Akt, IFG-1, and Bcl-2 (which is predominantly expressed in secretory FTE) (78). The estrogen dominant phase during the ovarian cycle is the follicular (or proliferative) phase and is associated with an increase in FTE proliferation (68) and promotion of ciliogenesis (83). In contrast, progesterone receptor activity is associated with a decrease in cell proliferation (68), an increase in apoptosis, possibly mediated through the down-regulation of CDK1/cyclin B1 complex, which impedes the G2/M transition. Conversely, in the breast, it is known that progesterone elicits proliferation through Cyclin D1 in PR positive cells (a cell intrinsic autocrine loop) and in PR negative cells via NF-κB ligand RANKL secretion (paracrine) (84, 85). Progesterone mediates its activity through the progesterone receptors (PR-A and PR-B are isoforms with differential translational start sites). On progesterone binding PR translocates to the nucleus to direct an antagonist effect on ERα signaling. Both ciliated and secretory FTE cells express the estrogen and progesterone receptors (69) and undergo cyclic changes in growth and differentiation throughout the ovarian cycle; these changes are most evident in the fimbriae (86) the “high-risk” zone of the tube (86). The fallopian tube epithelia in the luteal phase of the ovarian cycle have significantly lower levels of the progesterone (PR-A) (69) and estrogen [ERα) receptors (87)] (Figure 3).

During ovulation, there is a surge of estrogen released into the FTE microenvironment with release of follicular fluid, which contains high estrogen levels. This effect might be exacerbated (88) in fallopian tube epithelia of BRCA mutation carriers under the direct influence of the relevant DNA repair pathways, which are potentially dysfunctional. In addition to its well-established roles in regulation of DNA damage response, the Brca1 protein inhibits ERα transcriptional activity through direct action of BRCA1 and ERα proteins and down-regulation of p300, a nuclear receptor co-activator (89). Brca1 protein also regulates estrogen receptor action through suppression of aromatase, the enzyme required for estrogen biosynthesis from androgen. Gorrini et al. recently showed that an antioxidant estrogen target gene – Nrf2, can mediate a pro-survival effect in the absence of normal BRCA1 protein, in which cells would otherwise undergo cellular senescence or death (66). BRCA1 loss in mammary epithelium therefore alters the estrogenic growth response, and increased estrogen signaling collaborates with Brca1 deficiency to accelerate preneoplasia and cancer development. Although this has not been tested in FTE, this is an interesting concept that may have implications in serous carcinogenesis.

A decrease in the transcription and translation (by immunohistochemistry) of PR-A and PR-B were observed in the luteal phase of both BRCA mutation carriers and population control (69). PR gene signatures were identified in a subset of FTE cases in the luteal phase that had a similar profile to HGSC, however, PR target genes were not differentially expressed between BRCA mutation carriers and controls (69). In HGSC, PR expression is predominantly decreased/lost, a finding in 70–80% of patients (69, 90). PR expression in greater than 50% of tumor cells has been recently reported to have an overall survival benefit, and this benefit was independent of germline BRCA1/2 mutation status (90). In contrast, 70–80% of HGSC patients express ERα (>50%) but ER expression has not been shown to be associated with a significant recurrence free progression or survival benefit (87, 90).

Epidemiological data indicate that HGSC risk is closely linked to the events of ovulation, and these risk factors and protective factors for the most part are true for both sporadic and hereditary HGSC. In addition, evidence suggests that the risk for EOC increases during the pre-menopausal years, and that menopause is protective against ovarian cancer (91). The role of sex hormones in ovarian cancer development is complex however, and early evidence suggests that endocrine function may differ in BRCA1 heterozygotes. The mechanisms of altered hormone function and impact of genetic mutations on endocrine production and receptivity in the FTE of high-risk patients is not yet understood, but it remains possible that the underlying growth stimulatory effects of estrogen are altered in a BRCA mutation carrier.

## Concluding Remarks

In conclusion, there are many epidemiological studies linking ovulation, parity, and hormonal use to the development of EOC. About 60% or more of HGSC demonstrate a BRCAness profile predominantly through a dysfunctional homologous recombinant pathway, which synergizes with the ubiquitousness of the p53 mutations found amongst these tumors. In the normal fallopian tube of BRCA mutation carriers, transcription profiles reveal predominant differences in DNA damage and inflammation pathways. Interestingly and may be not surprisingly, FTE-BRCA samples are transcriptionally indistinguishable from FTE-normal samples when transcription profiles undergo unsupervised hierarchical clustering (70). Instead, the sample alignment is dependent on the estrogen or progesterone dominant phases of the ovulatory cycles, lending biological support to the known epidemiological risk factors and providing evidence for a possible haploinsufficiency of the functional allele in the normal FTE.

## Conflict of Interest Statement

The authors declare that the research was conducted in the absence of any commercial or financial relationships that could be construed as a potential conflict of interest.
